# Total ankle replacement: a population-based study of 515 cases from the Finnish Arthroplasty Register

**DOI:** 10.3109/17453671003685459

**Published:** 2010-03-31

**Authors:** Eerik T Skyttä, Helka Koivu, Antti Eskelinen, Mikko Ikävalko, Pekka Paavolainen, Ville Remes

**Affiliations:** ^1^COXA Hospital for Joint ReplacementTampere; ^2^Centre for Rheumatic Diseases, Department of Orthopaedics, Tampere University HospitalTampere; ^3^Rheumatism Foundation HospitalHeinola; ^4^Department of Orthopaedics and Traumatology, Surgical Hospital, Turku University HospitalTurku; ^5^Department of Musculoskeletal Medicine, Medical School, University of TampereTampereen Yliopisto; ^6^ORTON Orthopedic HospitalHelsinki; ^7^Department of Orthopedics and Traumatology, Peijas Hospital, Helsinki University Central Hospital, HUSFinland

## Abstract

**Background and purpose:**

Although total ankle replacement (TAR) is a recognized procedure for treatment of the painful arthritic ankle, the best choice of implant and the long-term results are still unknown. We evaluated the survival of two TAR designs and factors associated with survival using data from the nationwide arthroplasty registry in Finland.

**Methods:**

573 primary TARs were performed during the period 1982–2006 because of rheumatic, arthritic, or posttraumatic ankle degeneration. We selected contemporary TAR designs that were each used in more than 40 operations, including the S.T.A.R. (n = 217) and AES (n = 298), to assess their respective survival rates. The mean age of the patients was 55 (17–86) years and 63% of operations were performed in women. Kaplan-Meier analysis and the Cox regression model were used for survival analysis. The effects of age, sex, diagnosis, and hospital volume were also studied.

**Results:**

The annual incidence of TAR was 1.5 per 10^5^ inhabitants. The 5-year overall survivorship for the whole TAR cohort was 83% (95% CI: 81–86), which agrees with earlier reports. The most frequent reasons for revision were aseptic loosening of one or both of the prosthesis components (39%) and instability (39%). We found no difference in survival rate between the S.T.A.R. and AES designs. Furthermore, age, sex, diagnosis, and hospital volume (< 10 and > 100 replacements in each of 17 hospitals) did not affect the TAR survival.

**Interpretation:**

Based on our findings, we cannot conclude that any prosthesis was superior to any other. A high number of technical errors in primary TARs suggests that this low-volume field of implant arthroplasty should be centralized to fewer units.

## Introduction

Total ankle replacement (TAR) is a rather infrequently performed operation with a reported annual incidence of 0.7 per 10^5^ (Henricson et al 2007). Most TARs are implanted in end-stage rheumatoid arthritis (RA) but the proportion of other diagnoses has been increasing (Stengel et al. 2005, Fevang et al. 2007, Henricson et al 2007, Wood et al. 2008). Specialized centers have reported TAR 5-year survival rates of 70–93% (Anderson et al. 2003, Knecht et al. 2004, Spirt et al. 2004, Doets et al. 2006, San Giovanni et al. 2006, Wood et al. 2008, 2009) using second- and third-generation implants. The Norwegian and Swedish national registries have reported slightly inferior survival rates of 78–89% at 5 years and 62–72% at 10 years (Fevang et al. 2007, Henricson et al. 2007).

We analyzed the survival of two TAR designs and the factors affecting survival at a national level by using the data from the Finnish Arthroplasty Register.

## Patients and methods

Our study was based on information recorded in the Finnish Arthroplasty Register (Puolakka et al. 2001) relating to patients who underwent TAR between 1982 and 2006. The coverage of the Finnish Arthroplasty Register was analyzed in 1994–1995 by comparing its data with those of the discharge registers of the participating hospitals; it was found to cover 90% of implantations and implant removals. Since 1995, the data in the register have been compared with those of the hospital discharge registers every few years. Currently, over 95% of implantations are recorded. An English translation of the form used for data collection has been published elsewhere (Paavolainen et al. 1991). Revisions were linked to the primary operation using the unique personal identification number assigned to each resident of Finland.

There were data on 645 TARs, each of which have been recorded individually for every operation since the start of the register. Of these 645 TARs, 573 (89%) were primary operations and 72 (11%) were revisions.

### Inclusion criteria

In order to assess the survival of different TAR designs, we selected only those designs that had been used in more than 40 operations during the study period (Havelin et al. 1995). In addition, only implants with a mean follow-up of more than 2 years and more than 20 patients at risk at 3 years were included. We included bilateral TARs as separate cases in the analysis since bias by this procedure is probably negligible (Robertsson and Ranstam 2003).

### Type of prosthesis

To meet our inclusion criteria, the following implants were selected: LINK S.T.A.R. (Scandinavian Total Ankle Replacement) System (Waldemar Link, Hamburg, Germany) and BIOMET AES (Ankle Evolutive System) (manufactured by Transystème, Nîmes, France and distributed by Biomet, Valence, France). Both prostheses are third-generation mobile-bearing designs. All the S.T.A.R. prostheses were uncemented, double-coated versions. All the AES implants were uncemented, but the implant was changed by the manufacturer in 2004—from single-coated to a porous double-coated version with monobloc tibial component (the earlier tibia was modular). Of the other implant designs in use in Finland at the end of 2006, the Hintegra (Newdeal, Lyon, France) was excluded due to an insufficient number of operations (11). The use of the earlier designs was infrequent, and the designs were discontinued in the late 1980s (Table 1). 515 primary TARs were included in the study.

**Table 1.** Implants used for primary total ankle replacement in Finland during the period 1980–2006

Brand of implant	n	Used during years	Included in analysis
AES	298	2002–2006	yes
S.T.A.R.	217	1997–2006	yes
ICLH	32	1980–1987	no
Hintegra	11	2005–2006	no
Thompson-Parkridge- Richards	6	1980–1984	no
Oregon	4	1980–1985	no
RCM	3	1981–1984	no
Other	2	1980–1986	no
Total	573		

### Diagnosis

Half of the 515 TARs were performed due to rheumatoid arthritis (RA) (n = 252, 49%). Other indications included posttraumatic (n = 111, 22%) and primary (n = 99, 19%) osteoarthritis, other arthritides (n = 9, 2%), and other diseases (n = 44, 8%). We analyzed the overall survival of TARs performed due to RA and compared it to the “other indications” group in order to assess the effect of underlying disease.

### Hospitals

355 (69%) of the 515 TARs were performed in 1 foundation-based hospital and 2 university hospitals, each of which performed more than 100 TARs during the period 1997–2006 (group A). 4 more hospitals had performed 10–50 TARs (group B), and 10 other hospitals less than 10 operations (group C). We analyzed the overall survival of TARs performed in high-volume hospitals (group A) and compared this to that of the 2 low-volume hospital groups together (A vs. B and C) and separately (A vs. B vs. C) in order to assess the effect of hospital volume.

### Statistics

The endpoint for survival was defined as revision with either one component or the whole implant removed or exchanged. Kaplan-Meier survival data were used to construct the survival probabilities of implants at 1, 3, 5, and 7 years. Survival data obtained in the Kaplan-Meier analysis were compared by the log-rank test. The Cox multiple-regression model was used to study differences between groups and to adjust for potential confounding factors. In all models, the confounding factors were age and sex. The factors studied with the Cox model were TAR design and hospital type (high-volume hospitals vs. low-volume hospitals). All models included adjustment for differences in age and sex.

The Cox regression analyses provided estimates of survival probabilities and revision risk ratios (RR) for different factors. Estimates from the Cox analyses were used to construct adjusted survival curves at mean values of the risk factors. The Wald test was used to calculate p-values for data obtained from the Cox multiple-regression analysis. Differences between groups were considered statistically significant if the p-values were less than 0.05 in a two-tailed test. We used the SPSS software version 17.0.

## Results

### Patient characteristics

**Figure 1.**
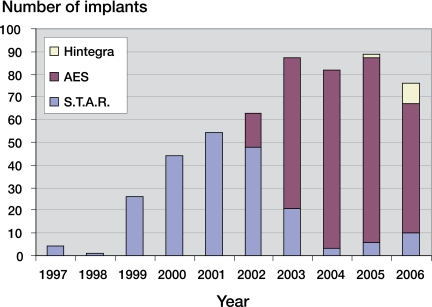
Number of ankle prostheses implanted per year in Finland during the period 1997–2006.

463 (90%) of the primary TARs were unilateral, and in 26 patients a 2-stage bilateral replacement was done. Of the 515 TAR operations, 327 (63%) were performed in women. At the time of the operation, the mean age of the patients was 55 (17–86) years. During the last 5 years, the mean annual incidence of TAR was 1.5 (95% CI: 1.3–1.8) per 10^5^ inhabitants (Figure 1).

### Implants

Over the whole study period, 9 TAR designs were used but only 2 of them had more than 40 operations (Table 1). At the end of the study period (2005–2006), 3 TAR designs were still being used.

### Disease-dependent survival

The Cox regression model (adjusted for age and sex) showed a similar risk of revision in all disease subgroups.

### Design-dependent survival

**Figure 2.**
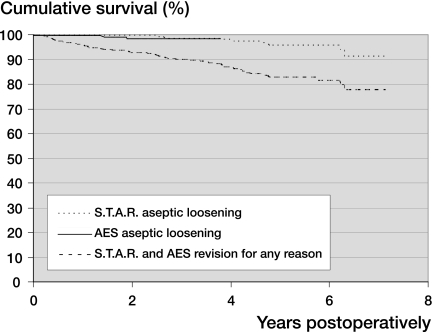
Kaplan-Meier survival curves for 217 S.T.A.R. and 298 AES total ankle replacements (with a mean follow-up of 4.8 and 2.0 years, respectively). The endpoint was defined as revision for aseptic loosening of one or both of the prosthesis components, and revision for any reason. The difference between survival rates for aseptic loosening of the 2 kinds of ankle replacements is not statistically significant (Log-rank test; p = 0.2).

**Figure 3.**
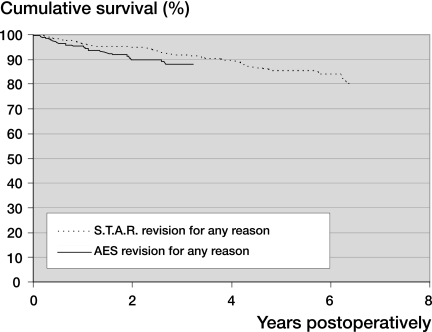
Kaplan-Meier survival curves for 217 S.T.A.R. and 298 AES total ankle replacements (with a mean follow-up of 4.8 and 2.0 years, respectively). The endpoint was defined as revision for any reason. The difference between survival rates is not statistically significant (Log-rank test; p = 0.08).

We found no differences in survival rates between S.T.A.R. and AES TAR designs over the whole study period, using either Kaplan-Meier analysis or the Cox regression model (with or without adjustment for age and sex) (Tables 2 and 3 and Figures 2 and 3). The 5-year survivorship for the whole TAR cohort was 83% (95% CI: 81–86) using revision for any reason as endpoint, and 95% (95% CI: 92–92) using revision for aseptic loosening as endpoint.

**Table 2.** Ankle replacements related to diagnosis during the period 1997–2006

	Rheumatoid arthritis	Other indications	All indications
Mean follow-up time in years (range)	3.4 (0.1–9.6)	2.9 (0.1–9.1)	3.2 (0.1–9.6)
Mean age at operation (range)	53 (17–81)	56 (18–86)	55 (17–86)
% Women	78%	50%	63%
Total number of			
TARs	252	263	515
S.T.A.R.	128	89	217
AES	124	174	298
No. of patients with bilateral operations	18	8	26

**Table 3.** Survival rates and Cox-adjusted risk ratios for revision of S.T.A.R. and AES total ankle replacements during the period 1997–2006 in Finland. The endpoint was defined as revision for any reason, and revision for aseptic loosening of one or both of the prosthesis components

A	B	C	D	E	F	G	H	I
Revision for any reason								
S.T.A.R.	31 / 217	4.8 (0–9.6)	199 96 (94–99)	180 92 (88–96)	112 85 (80–90)	24 80 (73–88)	1.0	–
AES	28 / 298	2.0 (0–4.7)	227 96 (93–98)	69 88 (84–93)	– –	– –	7 (0.9–3.0)	1. 0
Aseptic loosening								
S.T.A.R.	9 / 217	4.8 (0–9.6)	199 100 (99–100)	180 98 (97–100)	112 96 (93–99)	24 91 (84–98)	1.0	–
AES	5 / 298	2.0 (0–4.7)	227 100 (99–100)	69 98 (97–100)	– –	– –	2.7 (0.7–10.9)	0.2
A Implant design								
B No. of revisions / no. of total operations.								
C Mean follow-up in years (range)								
D At risk at 1 year, 1-year survival (%) and (95% CI)								
E At risk at 3 years, 3-year survival (%) and (95% CI)								
F At risk at 5 years, 5-year survival (%) and (95% CI)								
G At risk at 7 years, 7-year survival (%) and (95% CI)								
H Adjusted risk ratio from the Cox regression analysis (adjustment was made for age and sex) for revision (95% CI)								
I p-value								

### Age- and sex-dependent survival

Age and sex did not have any statistically significant effect on survivorship in the Cox multiple regression models (unadjusted and adjusted for implant design).

### Hospital volume-dependent survival

Hospital volume did not have any statistically significant effect on survivorship in the Cox multiple regression models (unadjusted and adjusted for implant design).

### Revision operations

During the period 1997–2006, 59 revisions were reported (Table 4). Thus, the 7-year survivorship for the whole TAR cohort was 78% (95% CI: 71–85). The most common reasons for revision were aseptic loosening (39%, n = 23) and instability (39%, n = 23). This was followed by primary malalignment of the prosthesis (8%, n = 5), infection (7%, n = 4), fracture of the meniscal implant (5%, n = 3), and periprosthetic fracture (2%, n = 1).

**Table 4.** Reasons for 59 revisions in 217 S.T.A.R. and 298 AES total ankle replacements (with a mean follow-up time of 4.8 and 2.0 years, respectively)

	S.T.A.R.	AES	All
Aseptic loosening	16	7	23
tibia	10	2	–
talus	3	5	–
both	3	0	–
Infection	3	1	4
Instability^**a**^	6	17	23
Fracture of the meniscal implant	2	1	3
Periprosthetic fracture	0	1	1
Primary malalignment of the prosthesis	4	1	5
Total	31	28	59
**^a^** With or without dislocation of the meniscal implant.			

## Discussion

Our main finding was the relatively low survival of TAR, with only 83% of cases revision-free at 5 years. We found no differences in survival between different TAR designs or concepts. Age, sex, diagnosis, and hospital volume did not affect the TAR survival. The mean annual incidence of TAR was 1.5 per 105, which is twice the reported incidence in Sweden (Henricson et al. 2007).

We acknowledge that our registry-based study has certain limitations. For example, we were not able to report any subjective outcome measurements, e.g. ankle performance scores or disease-specific quality of life measurements. Moreover, no radiographic analyses were done. Furthermore, in rheumatoid patients, a registry-based study may have pitfalls in that some of the patients diagnosed with RA are actually affected by juvenile or other subtypes of chronic arthritis. Another important issue, especially with an ankle register, is that a failed TAR is often converted to an arthrodesis. This may result in under-reporting of failures despite instructions, because the implants used in ankle arthrodesis are not reported to the arthroplasty register and a small number of these conversions may be done by orthopedic surgeons who are not familiar with reporting to the register.

In studies based on the Swedish and Norwegian national registries, aseptic loosening has been the most frequent reason for revision (31–48%) (Fevang et al. 2007, Henricson et al. 2007), results that are inferior to those of total hip and knee arthroplasty. Curiously, the survival rate reported here (95% using revision for aseptic loosening only as the endpoint) is similar to the survival of total knee arthroplasty (94–96%) (Furnes et al. 2002). Considering the major complications recently found with the AES implant as a result of osteolysis (Koivu et al. 2009), the currently good survival rates may become worse within the foreseeable future.

Interestingly, the proportion of revisions done for instability in primary TAR was notably high (39%) compared to the Swedish (16%) and Norwegian results (14%), which probably indicates a long learning curve for TAR. However, some of the primary TARs revised for instability may have been in acceptable alignment and balance immediately after surgery. In these cases, a marked preoperative varus or valgus deformity may have been corrected but degenerated ligaments may have been insufficient to sustain the balance after full weight bearing, and components may have migrated during follow-up due to poor bone quality, especially in RA. The migrated prosthesis components were found to be well-fixed in many of these cases at the time of revision.

It is commonly believed that the patient’s age, sex, and diagnosis have an influence on implant survival in TAR. In the Swedish study, only lower age was associated with an increased risk of later revision, but similarly to the Norwegian study we did not find such an association.

Jain et al. (2004) found a clear association between surgeon volume and outcome in patients who had a total shoulder arthroplasty or hemiarthroplasty. We have previously found an association between the hospital volume and failure in total elbow arthroplasty (Skyttä et al. 2009), and several studies have associated both surgeon volume and hospital volume with implant failure in hip replacement (Solomon et al. 2002). Fevang et al. (2007) found similar survival after TAR regardless of the size of the hospital. Henricson et al. (2007) found better 5-year survival in surgeons who had preformed more than 40 TARs, when comparing their first 30 TARs with operations performed thereafter. In our study, we did not find any differences in TAR survival when comparing high-volume and low-volume institutions. There are two explanations for this finding. First of all, many of the low-volume institutions are private clinics, where the surgeon comes from a high-volume hospital to perform the operation. Secondly, as the number of TARs performed annually in Finland is low, few surgeons ever reach and maintain the level of skill at the top of the learning curve.

There was an obvious shift from the S.T.A.R. prosthesis to the AES prosthesis during 2002–2003. The main reasons for this are technical: surgical instrumentation for the S.T.A.R. prosthesis is more difficult to use and the lack of trial implants caused many ankle surgeons to switch to the AES (Table 4). Also, the prosthesis sizing may be more favorable with the AES, allowing proper anteroposterior coverage without excess malleolar resection. Furthermore, radiographic follow-up of the S.T.A.R. talar implant is more complicated than with the AES.

Based on our findings, we cannot conclude that any prosthesis was superior to any other. A high number of complications in primary TARs were associated with balance and instability. These issues require a profound understanding of not only the ankle joint, but also the whole hindfoot. Based on earlier studies on low-volume joint replacement (Jain et al. 2004, Henricson et al. 2007, Skyttä et al. 2009), we suggest that performance of TARs should be centralized. With the Finnish population of 5.3 million, a sufficient number of TAR units would be 2–3 with 2 surgeons operating together at each unit. Thus, each surgeon would operate or assist at at least 25 TARs annually.
